# An Affordable, User-friendly Telerehabilitation System Assembled Using Existing Technologies for Individuals Isolated With COVID-19: Development and Feasibility Study

**DOI:** 10.2196/24960

**Published:** 2020-12-10

**Authors:** Masahiko Mukaino, Tsuyoshi Tatemoto, Nobuhiro Kumazawa, Shigeo Tanabe, Masaki Katoh, Eiichi Saitoh, Yohei Otaka

**Affiliations:** 1 Department of Rehabilitation Medicine I School of Medicine Fujita Health University Toyoake Japan; 2 Faculty of Rehabilitation School of Health Sciences Fujita Health University Toyoake Japan; 3 Department of Rehabilitation Fujita Health University Hospital Toyoake Japan

**Keywords:** telerehabilitation, COVID-19, telemedicine, isolation, user-friendly, feasibility, rehabilitation, eHealth

## Abstract

**Background:**

Isolation due to a COVID-19 infection can limit activities and cause physical and mental decline, especially in older adults and people with disabilities. However, due to limited contact, adequate rehabilitation is difficult to provide for quarantined patients. Telerehabilitation technology could be a solution; however, issues specific to COVID-19 should be taken into consideration, such as strict quarantine and respiratory symptoms, as well as accessibility to deal with rapid increases in need due to the pandemic.

**Objective:**

This study aims to develop and to investigate the feasibility of a telerehabilitation system for patients who are quarantined due to COVID-19 by combining existing commercial devices and computer applications.

**Methods:**

A multidisciplinary team has identified the requirements for a telerehabilitation system for COVID-19 and developed the system to satisfy those requirements. In the subsequent feasibility study, patients diagnosed with COVID-19 (N=10; mean age 60 years, SD 18 years) were included. A single session of telerehabilitation consisted of stretching exercises, a 15-minute exercise program, and a video exercise program conducted under real-time guidance by a physical therapist through a video call. The system included a tablet computer, a pulse oximeter, videoconferencing software, and remote control software. The feasibility of the system was evaluated using the Telemedicine Satisfaction Questionnaire (TSQ; 14 items) and an additional questionnaire on the telerehabilitation system (5 items). Each item was rated from “1 = strongly disagree” to “5 = strongly agree.”

**Results:**

The telerehabilitation system was developed by combining existing devices and applications, including a pulse oximeter and remote control mechanism, to achieve user-friendliness, affordability, and safety, which were determined as the system requirements. In the feasibility study, 9 out of 10 patients were able to use the telerehabilitation system without any on-site help. On the TSQ, the mean score for each item was 4.7 (SD 0.7), and in the additional items regarding telerehabilitation, the mean score for each item was 4.3 (SD 1.0).

**Conclusions:**

These findings support the feasibility of this simple telerehabilitation system in quarantined patients with COVID-19, encouraging further investigation on the merit of the system’s use in clinical practice.

## Introduction

Despite global efforts to mitigate the spread of COVID-19, which was declared a global pandemic by the World Health Organization on March 12, 2020 [1], the virus is showing no signs of slowing down. To counteract the rapidly spreading infection, many individuals, including those already infected by the virus as well as older adults and people with disabilities who are at high risk of severe pneumonia due to COVID-19, are quarantined to avoid contact. However, isolation limits activities and may cause physical and mental decline, particularly among older adults and people with disabilities [2].

Providing rehabilitation by alleviating the functional decline experienced by individuals in isolation due to COVID-19 should be a solution to this issue [3]. However, there have been concerns that rehabilitation typically involves human interaction at close proximity and physical contact, and therefore increases the risk of infection transmission [4].

Telerehabilitation may potentially address this problem. Recent developments in digital technology have made it possible to conduct telerehabilitation using real-time communication technology [5]. However, there are several issues in applying telerehabilitation for patients with COVID-19. For example, there is an issue around operation of the systems by the patients who are quarantined. In applying telerehabilitation to patients, proficiency in the operation of videoconferencing systems, monitoring devices, and applications is necessary because the patients have to operate them alone when using the system in their own room. In many previous studies in telerehabilitation, patients were given an opportunity to practice the exercise and operation under the supervision of therapists prior to the start of telerehabilitation [6-8]. However, this method cannot be applied to patients with COVID-19 because they are already quarantined when they start the telerehabilitation program.

Another issue may be the cost for the system. Previous studies have shown the feasibility and effectiveness of telerehabilitation with older adult patients and patients with disabilities using dedicated videoconferencing systems prepared for telerehabilitation [6-8]. Although those dedicated telerehabilitation systems have been shown to be effective, the initial investment for such systems may not be prioritized because of the rapid expansion in demand for medical resources caused by the COVID-19 pandemic. Therefore, there may be demand for a system that is affordable and accessible for immediate response to this pandemic.

With the aim of solving these problems and providing exercise opportunities for patients who are isolated, we developed and tested the feasibility of a simple telerehabilitation system using common, commercially available devices and applications.

## Methods

### Participants

Patients who were diagnosed with COVID-19 and admitted to a university hospital, and who agreed to participate in this study were included. The exclusion criteria were as follows: requirement of oxygen therapy, existence of hearing loss, existence of severe orthopedic or neurologic disease, and inability to understand instructions. The patients’ demographics, including the days after onset, the severity of the pneumonia at its worst (severe: requiring incubation; moderate: requiring oxygen; mild: presenting signs of pneumonia but do not require oxygen; and asymptomatic), and comorbidities, were collected from the medical record. The levels of functioning of the patients were evaluated using the Functional Independence Measure (FIM) [9]. This study is approved by the institutional review board. All patients provided written informed consent prior to study participation.

### Telerehabilitation System

The telerehabilitation system was prepared in private rooms where the patients were quarantined in the university hospital. The telerehabilitation system was designed and built by a team consisting of two physicians, an engineer, and two physical therapists. The preliminary version of the system was introduced previously [10]. The team first discussed and defined the requirements for the telerehabilitation system for COVID-19 and then developed the system to satisfy the requirements. The hardware of the system consists of a desktop computer for the health professionals who are providing the service, a tablet computer for patients, and a pulse oximeter to monitor vital signs. The pulse oximeter is a commercially available product (RingO2, Neuroceutical Inc) that can be connected to a tablet via Bluetooth. On the tablet, the remote-controlled software TeamViewer (TeamViewer GmbH), conferencing softwares Zoom (Zoom Video Communications Inc) and Skype (Microsoft Corp), and a pulse oximeter control application were installed. A video file of an exercise program was also preinstalled on the tablet computer in the patients’ rooms in case of an unstable internet connection. The cost for implementation is the price in Japan, with the value converted into US dollars (US $) and Euros (€) at the exchange rate on May 8, 2020, when the study started.

### Procedure

**Figure 1:**
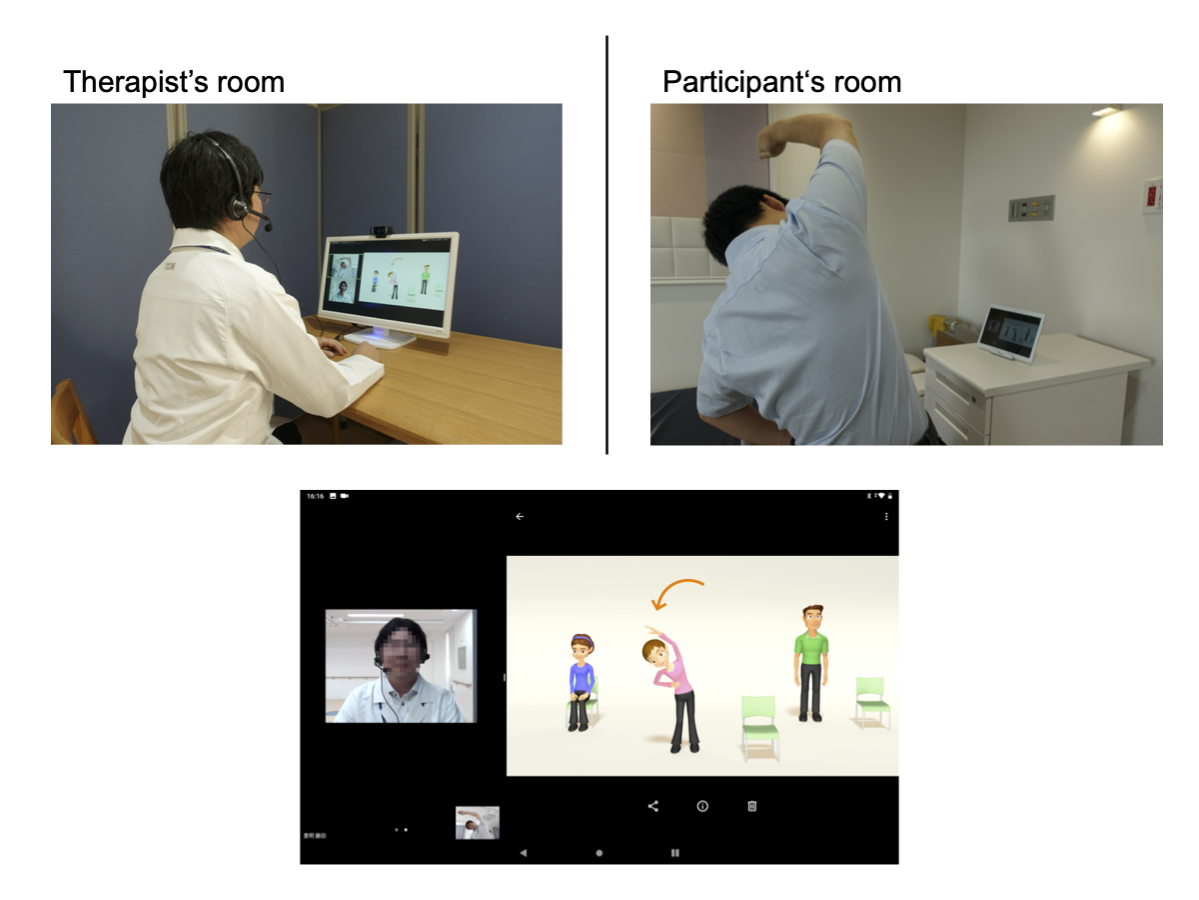
Exercise program delivered using the telerehabilitation system. Upper panel: A physical therapist instructs the patient about an exercise program using the telerehabilitation system. Lower panel: Screen of the tablet computer installed in the participant’s room.

The exercise program was performed in the following manner (Figure 1). A physical therapist with 12 years of professional experience provided the service. First, at a prescheduled time, the physical therapist called the patient via Skype and instructed them to start the TeamViewer software to enable remote control of the tablet from the therapist’s side. The therapist then launched the apps (Zoom and the app for controlling the pulse oximeter) to start the exercise. The vital signs and basic motor ability were checked, and then, a video exercise program was performed. After ending the program, the vital signs were checked again. The total length of the session was 20 minutes including preparation. The patients received the survey on the feasibility of the program after a single session.

### A Survey for Feasibility of the Program

A survey using the Telemedicine Satisfaction Questionnaire (TSQ) [11] was administered after the end of the program. The TSQ was developed by Yip et al [11] to assess satisfaction with telemedicine, and it consists of 14 items and a 5-point Likert scale ranging from 1 (strongly disagree) to 5 (strongly agree).

In this study, the following five questions were added to further assess satisfaction with the delivery of exercise programs using the system: “I can easily understand how to move,” “I feel safe performing the exercise,” “The room environment is appropriate for performing the exercise programs,” “The devices used are appropriate for performing the exercise programs,” and “Telecommunication with medical experts during exercise is helpful.” The changes in SpO_2_ levels before and after the exercise were also evaluated.

## Results

### Development of the System

As a result of the discussion on system requirements by a multidisciplinary team, three requirements were set for the system: (1) user-friendliness, to be used without prior practice because patients with COVID-19 are isolated from the beginning; (2) affordability, to be quickly implemented in response to the rapid spread of COVID-19 infection; and (3) safety, achieved through the real-time monitoring of vital signs to be able to respond to a possible rapid deterioration in respiratory function during exercise. The system specifications were determined and developed as follows to satisfy each of the predefined requirements:

1. User-friendliness: The lack of practicing in advance was expected to cause significant problems in operating the devices in patients with low digital literacy. Therefore, the system was developed to let the service provider operate most of the functions on behalf of the patient using a remote control application (TeamViewer). Since the content of the exercises could not be practiced in advance, we prepared a video for a 15-minute exercise program as a tool to assist in teaching the exercises. The video of the exercise program was preinstalled on the patient’s tablet PC to avoid the quality of playback being affected by the quality of the internet connection. During the exercise, the video was started remotely by the service provider, and the exercises were performed along with the video, with real-time advice by the therapists.
2. Affordability: In this regard, the system was created using existing devices and applications, with an emphasis on being inexpensive and readily available. The hardware was simple, consisting of a PC on the service provider’s side and a tablet PC on the patient’s side. Zoom and Skype, which are common videoconferencing applications, and TeamViewer, a remote control application, were installed on the tablet. As a monitoring device of vital signs, a pulse oximeter (RingO2), which can be connected to the tablet PC via Bluetooth, was prepared. The initial cost for preparation of the system for a single set of hardware and software was ¥287,800 (US $2705.90; €2489.41) for the service provider and ¥39,800 (US $383.41; €344.27) for a single patient. The details of the cost are shown in Table 1.
3. Safety: To address this point, a pulse oximeter was prepared for real-time monitoring during the exercise to respond to possible difficulty in respiratory function. A pulse oximeter (RingO2) that can be connected to a tablet PC via Bluetooth was used. The service provider could check the blood oxygen saturation and heart rate of the patients through the screen during the session.

**Table 1:** Detailed initial costs for the devices and applications.

Items and products				Provider		Cost (¥)		Cost (US $)^a^		Cost (€)^b^
**Service provider kit**										
	**Laptop PC**									
		MacBook Pro 13 inches	Apple Inc		188,800		1775.10		1633.08	
	**Remote control software**									
		TeamViewer Business license	TeamViewer GmbH		59,400 (per year)		558.48		513.80	
		TeamViewer mobile devise support option	TeamViewer GmbH		39,600 (per year)		372.32		342.53	
	**Conferencing software**									
		Zoom Meetings (Basic license)	Zoom Video Communications Inc		Free		Free		Free	
		Skype	Microsoft Corporation		Free		Free		Free	
	Total (service provider kit)			N/A^c^		287,800		2705.90		2489.41
**Patient kit**										
	**Tablet PC**									
		LAVIE tablet PC	NEC Corporation		20,000		188.04		173.00	
	**Pulse oximeter**									
		RingO2 (with viewer application)	Neuroceuticals Inc		19,800		186.16		171.27	
	Total (patient kit)			N/A		39,800		374.20		344.27

^a^US $ was calculated from ¥ at the market price as of May 8, 2020: 106.36 ¥/US $

^b^€ was calculated from ¥ at the market price as of May 8, 2020: 115.61 ¥/€

^c^N/A: not applicable.

### Feasibility Study

A total of 10 patients with COVID-19 participated in this study. The demographic variables of the patients are summarized in Table 2. The mean age of the patients was 60 (SD 18) years; 4 patients were men, and 6 were women. The average SpO_2_ levels at the start and end of the study were 94.4 (SD 2.6) and 95.1 (SD 2.0), respectively. There were 3 patients that experienced moderate pneumonia, 5 had mild signs of pneumonia, and the others were asymptomatic. The average FIM of the patients was 122.4 (SD 4.5; mean motor score 87.7, SD 4.3; mean cognitive score 34.4, SD 1.1). Out of the 10 patients, 4 had no experience using a tablet PC or smartphone.

**Table 2:** Patient demographics.

Demographic		Patients (N=10)
Age (years), mean (SD)		60 (18)
**Gender, n**		
	Male	4
	Female	6
**Pneumonia at its worst, n**		
	Moderate	3
	Mild	5
	Asymptomatic	2
Days after diagnosis, mean (SD)		9.9 (8.0)
**Comorbidities, n**		
	Hypertension	4
	Diabetes	2
	Stroke	1
	Mild cognitive impairment	1
	Depression	1
	Lumbar compression fracture	1
**Functional Independence Measure, mean (SD)**		
	Total score^a^	122.4 (4.5)
	Motor score^b^	87.7 (4.3)
	Cognitive score^c^	34.4 (1.1)
**Experience in using tablet PC or smartphone, n**		
	Yes	6
	No	4

^a^Range of total score was 18-126.

^b^Range of motor score was 13-91.

^c^Range of cognitive score was 5-35.

All the patients successfully completed the session. The average time required for preparation after the first Skype call to the start of the exercise was 3.0 (SD 1.1) minutes. In 1 case out of 10, on-site support for handling tablet computers was required at the start of the session. The patient had no cognitive disorder, had been treated for depression, and had no experience using a tablet PC. The patient asked for on-site help because of an unexpected movement of the application at the start of the session. After the initial set up, the patient required no further on-site help.

**Figure 2:**
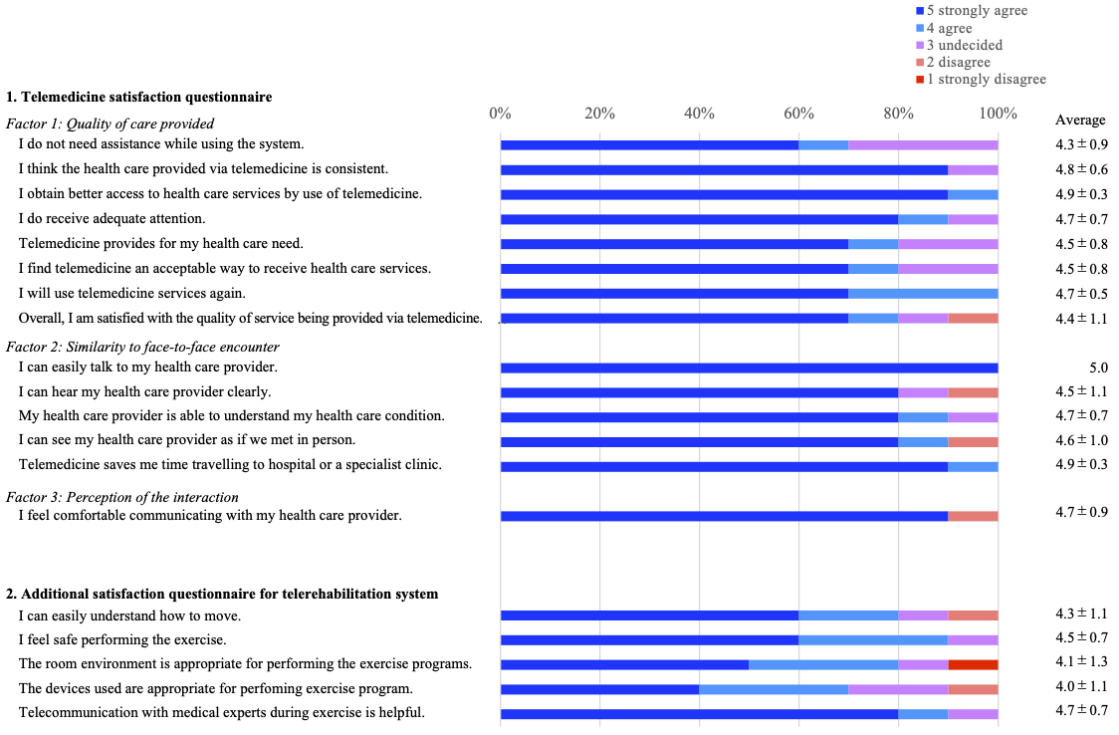
Telemedicine Satisfaction Questionnaire and additional questionnaire for the telerehabilitation system. The percentage of response options for each question is shown.

The results of the TSQ and the additional questions are shown in Figure 2. On the TSQ, the mean score for each item was 4.7 (SD 0.7), and the mean total score of the TSQ (maximum score of 70) was 65.2 (SD 6.9; mean 93.1%, SD 9.8%). In the additional items regarding telerehabilitation, the mean score for each item was 4.3 (SD 1.0). Additionally, there were several feedbacks from the participants regarding difficulty experienced while handling tablets and the limited visibility of video calls and video exercise programs. The TSQ was also administered to physical therapists who were service providers, with a total score of 61 (87.1%) and a mean score of 4.4 (SD 0.6).

## Discussion

### Principal Findings

In this preliminary study, a telerehabilitation system for patients isolated due to COVID-19 was developed by combining commercially available devices and applications, and its feasibility was tested in 10 patients with COVID-19. The exercise program was safely conducted, with no significant decrease in SpO_2_. Patients’ satisfaction with the telerehabilitation system was generally high.

Various telerehabilitation programs have been used in patients with orthopedic, neurological, respiratory, and cardiovascular diseases, and have been shown to be effective [12-15]. Telerehabilitation is considered to have potential benefits for patients, such as better accessibility, reduced time burden, and cost effectiveness [16-18]. It has also been shown that it may be as effective as on-site rehabilitation [6-8]. The use of telerehabilitation systems in isolated patients due to infection has rarely been reported, but the rapid spread of COVID-19 infections has increased interest in this area. The capabilities required for a telerehabilitation system for patients in isolation due to infection may differ from those of previous telerehabilitation systems.

In this study, we first identified three prerequisites (user-friendliness, affordability, and safety) as necessary for the establishment of a telerehabilitation system for patients in isolation due to infection, and then, we developed a system based on those prerequisites. User-friendliness is an important point in telerehabilitation [19], but it is particularly important in the case of telerehabilitation for infectious diseases. Many previous studies of telerehabilitation have consisted of prior practice and subsequent tele-sessions [6-8]. However, when practice is initiated in isolation from the beginning, the user needs to handle the device and applications, and perform exercise without any on-site assistance. Therefore, a high level of user-friendliness is necessary to successfully conduct the exercise sessions with patients that have variations in their levels of digital literacy.

Affordability and safety were also important, although they may conflict with user-friendliness. For example, the surest way to make the telerehabilitation system user-friendly is to create a customized system for the target patients. However, the development of customized systems may reduce affordability. In addition, monitoring is necessary for patient safety, which would complicate the system. To satisfy all these requirements, a new approach was taken in this study to combine existing devices and applications, including monitoring devices, and to minimize patient manipulation by the use of remote control.

The results of our study support the main assumed benefit of the system: user-friendliness. Out of the 10 patients in this study, 9 were able to complete the session without on-site assistance, and 7 patients agreed or strongly agreed with the item “no assistance is required while using the system.” Overall, a high level of satisfaction was observed, with the TSQ scores averaging 93.1% (65.2/70), which is comparable to previous studies [20-22]. However, there was some feedback from patients about usability issues, and further improvement in the user-friendliness of the system would help for widespread clinical implementation in the future. The effectiveness of the system should also be tested to see if this telerehabilitation system can contribute to the maintenance of function in isolated patients.

Another advantage of the system demonstrated in this study is that it was built using existing commercial resources and requires minimal initial cost. In fact, the initial costs required in this study were less than in previous studies [23,24], with a total of US $2781.12 for the service provider kit (including annual license of the applications) and US $383.41 for the patient kit. Many of the commercialized devices and applications such as PCs, tablet PCs, pulse oximeters, videoconferencing applications, and remote control applications used in this study already exist and can be prepared inexpensively. In addition, these resources are easily accessible, and therefore, the system can be easily replicated and deployed. Such an approach also eliminates the need to spend time and cost on developing the dedicated devices or apps, supporting the rapid social response to changes in the rehabilitation practice due to the COVID-19 pandemic.

### Limitations

There are several limitations. This study was conducted as a feasibility study with a small sample. The merit of conducting the telerehabilitation with this system on the patients’ functioning should be further investigated with a larger sample and a longer-term intervention.

Although the results of the TSQ support the good feasibility of this telerehabilitation system, it can be affected by the environment due to internet connection; these results were, in fact, obtained in a stable internet environment with a Wi-Fi connection. However, the system is designed to reduce the influence of unstable internet connection by preinstalling the video program on the tablet, so a level of internet speed that is stable enough to make video calls should be sufficient to provide the same quality of intervention as in this study.

A survey of service providers was also conducted, but only 1 person provided the service in this study, and there was insufficient information on the user-friendliness for the service side. In particular, because the service provider took over all operations on the patient’s side, the service provider’s operations have become more complicated, and there is room for improvement of the operation method and consideration of how to operate the system, such as the development of robotic process automation software.

### Conclusions

The telerehabilitation system developed in this study may be applicable to individuals experiencing isolation related to the COVID-19 pandemic. The results of this study should prompt further investigation of the usefulness of telerehabilitation in clinical settings.

## Abbreviations

FIM: Functional Independence Measure
TSQ: Telemedicine Satisfaction Questionnaire
